# Challenges with the use of Xpert HPV as a screening tool for oral HPV among people living with HIV (PLHIV): experiences from Pune, India

**DOI:** 10.1186/s12879-023-08210-2

**Published:** 2023-04-17

**Authors:** Abigail Admase, Samir Joshi, Rohidas Borse, Prasad Deshpande, Vandana Kulkarni, Samir Khaire, Rahul Thakur, Amol Chavan, Smita Nimkar, Vidya Mave, Ivan Marbaniang

**Affiliations:** 1 Zanvyl Kreiger School of Arts and Sciences, Johns Hopkins University, Baltimore, MD USA; 2 Department of Otorhinolaryngology and Head & Neck Surgery, Byramjee Jeejeebhoy Government Medical College, Pune, India; 3 Department of Medicine, Byramjee Jeejeebhoy Government Medical College, Pune, India; 4 Byramjee Jeejeebhoy Government Medical College - Johns Hopkins University Clinical Research Site, Pune, India; 5 Department of Dentistry, Byramjee Jeejeebhoy Government Medical College, Pune, India; 6 Center for Infectious Diseases in India, Johns Hopkins School of Medicine, Baltimore, MD USA; 7 Department of Epidemiology, McGill University, McGill College, Suite 1200, Montreal, QC Canada

**Keywords:** HPV, HIV, India, Xpert HPV

## Abstract

**Background:**

People living with HIV (PLHIV) are at higher risk for human papillomavirus (HPV)-related oropharyngeal cancers compared to the general population. Xpert HPV test is a polymerase chain reaction (PCR) assay capable of rapid HPV detection. Performing the assay requires minimal intervention by laboratory personnel. Its use could improve oropharyngeal cancer screening among PLHIV living in low-and middle-income countries (LMICs) with limited diagnostic capacities. However, Xpert HPV performance for oral samples has not been evaluated. Here, we describe our experience with Xpert HPV and compare its results with traditional PCR, for oral samples.

**Methods:**

Oral samples from 429 PLHIV receiving care at a tertiary care hospital affiliated antiretroviral therapy center in Pune, India were used. Samples were collected either after a 30s oral rinse and gargle (n = 335) or in combination with cytobrush scraping of the oral mucosa (n = 91). Unsuccessful tests were those that generated an invalid or error result on Xpert HPV. Successful tests were those that generated a positive or negative result. Kappa statistic was used to compare concordance between Xpert HPV and traditional real-time PCR results.

**Results:**

There were 29.8% (n = 127) unsuccessful tests, of which 78.7% (n = 100) were invalid and 21.3% (n = 27) were error results. Adding cytobrush scraping to oral rinse as a collection procedure did not significantly reduce the proportion of unsuccessful tests (p = 0.9). For successful tests, HPV positivity on Xpert was 0.3% (n = 1/299). Kappa statistic was 0.11, indicating poor agreement between Xpert HPV and traditional PCR results.

**Conclusions:**

Presently, Xpert HPV appears to have limited use for oral HPV detection among PLHIV using oral samples. More research to improve the diagnostic capabilities of Xpert HPV for oral samples among PLHIV is needed.

**Supplementary Information:**

The online version contains supplementary material available at 10.1186/s12879-023-08210-2.

## Introduction

People living with HIV (PLHIV) have 2-3-fold higher odds of prevalent oral human papillomavirus (HPV) compared to HIV-uninfected individuals [1]. Oncogenic high-risk HPV (hrHPV) types (mostly HPV16, HPV18 and HPV33) [2], have been isolated in 12–26% of oral samples of PLHIV in previous studies [1]. This puts PLHIV at an increased risk of oropharyngeal squamous cell carcinomas (OPSCCs), including cancers of the base of tongue, lingual and palatine tonsils [1, 2].

Traditional diagnostic methods for oral HPV rely on polymerase chain reaction (PCR) or in-situ hybridization (ISH) techniques [3]. These techniques require skilled laboratory personnel and several hours to complete [4]. In low-and middle-income countries (LMICs) where the dual burden of HIV and OPSCCs is high [5–7], trained personnel are lacking, and health systems congested, the feasibility of these techniques may be limited [5].

The Xpert HPV (Cepheid Sunnyvale, CA) test is a rapid, PCR assay for the qualitative real time detection of 14 types of hrHPV DNA [8]. Sample extraction, PCR amplification, and HPV detection are fully automated on Xpert HPV. The assay is performed on Cepheid GeneXpert instrument system. Positive qualitative findings can further be reported as positive or negative for HPV 16, HPV 18/45, and a pooled result for 11 hrHPV types (31, 33, 35, 39, 51, 52, 56, 58, 59, 66 and 68). The assay contains two internal controls, a Sample Adequacy Control (SAC) and a Probe Check Control (PCC). SAC reagents detect the presence of a single copy human gene and monitor the adequacy of human cells to carry out a qualitative HPV assessment. Failure of the SAC leads to an *invalid* result. The PCC verifies reagent rehydration, PCR tube filling, probe integrity, and dye stability. Failure of the PCC leads to an *error* result [8]. Running the assay requires minimal training and the instrument provides results within one hour, allowing for same day HPV screening [4, 9]. In LMICs, Xpert HPV could bolster oral cancer screening strategies by rapidly identifying PLHIV with oral hrHPV, who may benefit from same-day HPV counselling, closer clinical examination and follow-up.

Xpert HPV has been successfully deployed with acceptable validity for HPV detection among women living with HIV using cervical fluid samples [4], and more recently anal fluid samples [10, 11]. It has also been used for HPV detection in formalin-fixed paraffin-embedded oropharyngeal cancer samples [12]. However, the use of oral samples has not been evaluated. There is also limited evidence on the implementational challenges of Xpert HPV use. Previous studies of Xpert MTB/RIF assays for *Mycobacterium tuberculosis* have reported different proportions of invalid and error results globally [13–15], limiting the optimal use of the assay.

In this manuscript, using data collected from PLHIV in Pune, India our objectives are, (1) To describe the implementational challenges of HPV Xpert using oral samples, particularly SAC and PCC failures; (2) To assess the agreement of Xpert HPV with traditional PCR results for oral samples.

## Method

### Study population

We used data from a parent cross-sectional study that sought to evaluate the prevalence of oral potentially malignant disorders (OPMDs) among PLHIV (n = 601) and HIV-uninfected individuals (n = 633) [16]. For this analysis, we excluded HIV-uninfected individuals. Brief recruitment procedures for PLHIV described below were those followed in the parent study.

PLHIV were enrolled from the antiretroviral therapy (ART) center of Byramjee Jeejeebhoy Government Medical College – Sassoon General Hospitals (BJGMC-SGH) in Pune, a city in western India. The ART center caters to approximately 5000 PLHIV, belonging to lower and middle socioeconomic status. A registry-based study showed that the 28-to-60-month standardized incidence ratio of oral and oropharyngeal cancers was 27 times higher (95% CI: 19.7, 36.1) among PLHIV compared to the general population of Pune [17].

All PLHIV ≥ 21 years with no prior history of oral cancer attending the ART center between June 2017 and June 2019 were approached by two study counselors. Eligible participants that provided informed consent were enrolled into the study. Enrollees completed counsellor-administered questionnaires, provided oral samples and photographs of the oral cavity. Study procedures have been described at length elsewhere [16]. Sociodemographic, tobacco and alcohol use, sexual history, CD4 count, suspected OPMDs, Xpert HPV results, and traditional PCR results data were extracted from the database of the parent study.

The study was approved by the Ethics Committee of BJGMC-SGH and the Institutional Review Board of Johns Hopkins University (JHU).

### Laboratory methods

All laboratory procedures (unless explicitly stated) were carried out at the JHU-Clinical Research Site (CRS) laboratory associated with BJGMC-SGH. The laboratory is approved by the Division of AIDS, United States National Institutes of Health (NIH) and was involved in an NIH-funded HPV-related clinical trial (A5282) [18].

### Procedures for oral sample collection and processing

**Fig. 1:**
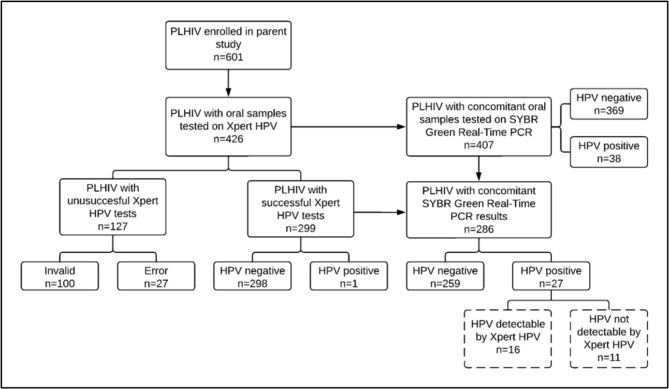
Flowchart showing the number of participant samples processed on Xpert HPV and traditional PCR respectively. Xpert HPV identifies only certain high risk HPV types (HPV 16, 18/45, 31, 33, 35, 39, 51, 52, 56, 58, 59, 66, 68). Therefore, even under perfect agreement with traditional PCR, Xpert HPV would have deemed positive only 16 of the 27 PCR HPV positive samples. Other low risk HPV types detected by conventional PCR (HPV 7, 13, 27, 32, 42, 44, 72, 90, 107, 120) would have been deemed negative by Xpert HPV (represented by dotted boxes)

Due to procurement delays for Xpert HPV supplies, we were able to test only 426 oral samples from PLHIV using the assay (Fig. 1). The procedures for sample collection and processing are described below. Most samples (n = 403) were collected between 9 am and 12 pm.

#### Procedure 1

##### Collection

For the first 335 participants, a 15 mL oral rinse sample was collected by instructing participants to alternate between swishing and gargling with alcohol-free Colgate® Plax mouthwash for 30 s. Samples were collected aseptically in a 50 mL Falcon™ conical tube (Becton Dickinson) and kept on ice until processed within four hours of collection.

##### Processing

The collected sample was centrifuged twice, at 5000 RPM and 4 degrees Celsius for 10 min each time. A 2 mL final suspension in Cervi-Collect solution (Abbott Molecular) was created using the pellet obtained after two centrifugations.

#### Procedure 2

##### Collection

The next 91 participants were first instructed to rinse their oral cavity with water and wait for 30 min. This was done to remove any particulate matter (particularly betel quid or tobacco residues) from the oral cavity. Using a cytobrush (Abbott Molecular), participants were then instructed to scrape their right and left buccal mucosa and the surface of any suspected OPMD. The cytobrush scraping was placed in Cervi-Collect solution. This was followed by an oral rinse as for Procedure 1.

##### Processing

A suspension using the oral rinse (as for Procedure 1) was created first. A final pooled suspension (2.5 mL) was created by vortexing the oral rinse suspension and the Cervi-Collect solution in which the cytobrush scraping was placed.

**Fig. 2:**
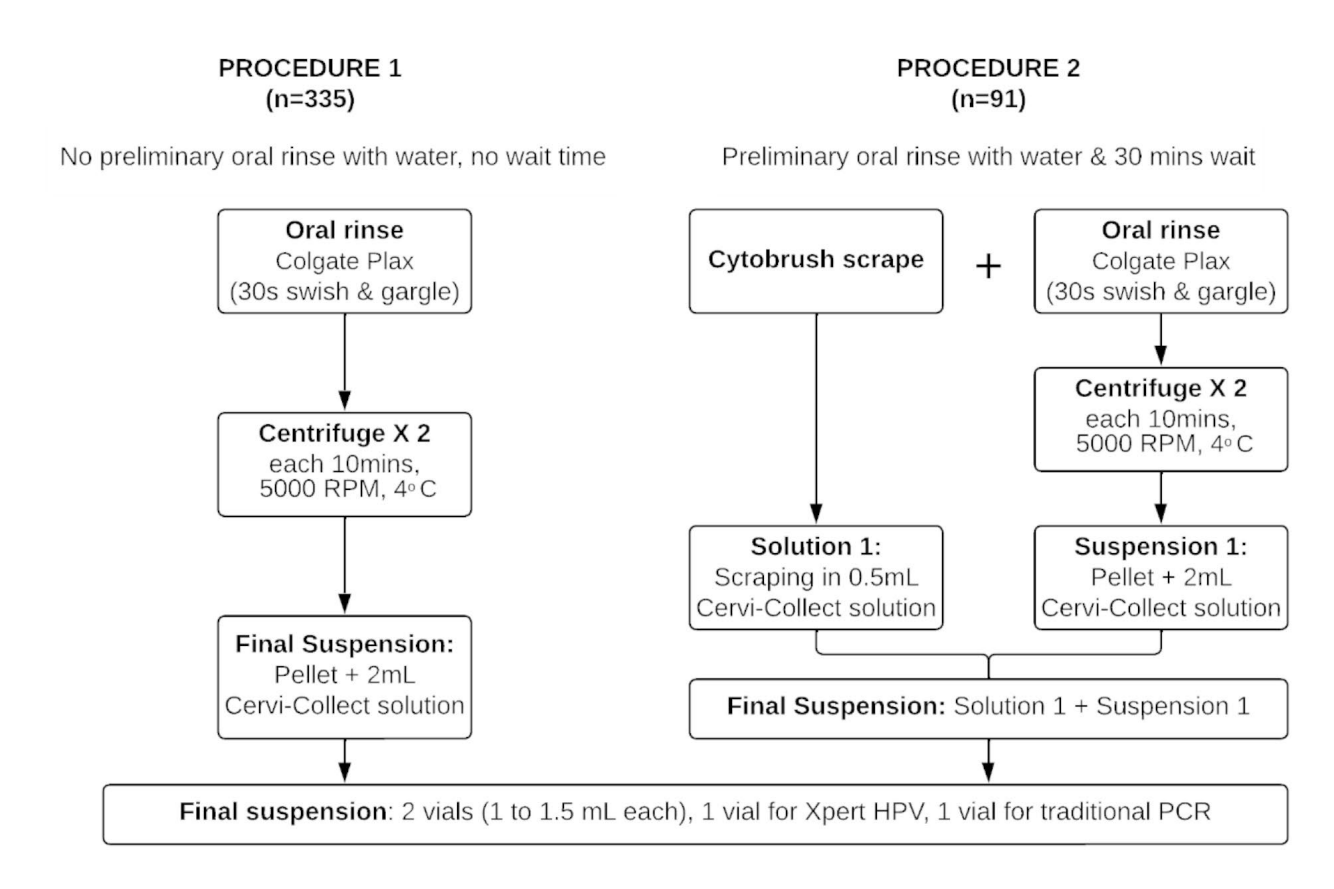
Schematic showing the collection and processing procedures used for oral samples

Final suspensions obtained from each procedure were divided into two vials, each of 1 to 1.5 mL **(**Fig. 2**).**

For the Xpert HPV assay, 1 mL (recommended volume) of the final suspension was used [8]. All samples were processed on the day of collection. Suspensions were stored at negative 80 degrees Celsius if not tested immediately. All testing using Xpert HPV assay was performed within two weeks of sample collection.

We changed from Procedure 1 to Procedure 2, because there were many Xpert invalid (n = 78) and error (n = 21) results for Procedure 1 (i.e., SAC or PCC failures). After consulting with experts at the NIH funded Virology Quality Assurance (VQA) program at Rush University, Chicago, USA [19], the addition of cytobrush scraping was recommended to mimic cervical sample collection methods more closely [9]. It was hypothesized that this may reduce SAC failures by increasing epithelial cell concentration of the final suspension. Simultaneously we posited that potential oral particulate matter could be a source of interference leading to invalid and error results. Therefore, a preliminary oral rinse with water was also included in Procedure 2, consistent with a recommendation to reduce the proportion of unsuccessful tests on Xpert MTB/RIF [20].

### Procedures to limit SAC and PCC failures

We undertook several steps to limit SAC and PCC failures. Prior to the start of the parent study, to verify that internal controls were functioning optimally, we pilot tested eleven Xpert HPV assays using a quality control sample for HPV created by VQA and 10 Xpert HPV assays using oral samples (utilizing Procedure 1). Pilot testing was conducted under the supervision of a Cepheid representative. There were no invalid or error results for these preliminary 21 samples. To reduce the variability in the instructions provided to participants all sample collections were supervised by the same two trained laboratory technicians. Further, to reduce variability in sample preparation, all centrifugations, suspension preparations, and assay runs (including pipetting 1mL of the final suspension into the Xpert cartridge) were conducted by the same technicians, supervised by a virologist trained in Xpert methodology. (Table 1)

**Table 1:** Internal quality control features of the Xpert HPV and steps undertaken by the laboratory at Byramjee Jeejeebhoy Government Medical College – Johns Hopkins University Clinical Research Site (BJGMC-SGH CRS) to reduce control failures

Control Step	Brief explanation of the control step	Test result displayed (if control fails)	Steps undertaken at BJGMC-SGH CRS to reduce control failure
Probe Check Control (PCC)	The PCC verifies reagent rehydration, PCR tube filling in the cartridge, probe integrity, and dye stability	Error	• Pilot conducted for 21 Xpert HPV assays (11 using quality control samples created by VQA + 10 using oral samples) prior to using the assays in the parent study. Pilot conducted under the supervision of a Cepheid representative • Adequate sample volume (i.e., 1ml) ensured for all tests • All testing conducted by two laboratory technicians supervised by a virologist trained in Xpert HPV methodology
Sample Adequacy Control (SAC)	SAC reagents detect the presence of a single copy human gene and monitor whether the specimen contains adequate numbers of human cells to carry out qualitative assessment of HPV status	Invalid	• Two trained laboratory technicians collected all samples to reduce variability in instructions provided to participants and performed all assays • Method of sample collection changed from oral rinse only to cytobrush scraping + oral rinse to increase exfoliated cell yield • Consultation with VQA before changing collection procedure

Table 1 has been adapted from The Public Report of the WHO Prequalification of In Vitro Diagnostics for Xpert HPV (version Decemeber 3rd, 2020); VQA: US National Institutes of Health (NIH) funded Virology Quality Assurance program at Rush University, Chicago, USA

All tests were conducted in an air-conditioned laboratory, where room temperature was charted regularly and maintained at or below 25 degrees Celsius. There were no interruptions to electricity supply, and all assays were stored at 20 degrees Celsius (recommended 2 to 28 degrees Celsius). The Xpert instrument was kept at 15 cm from the wall to allow any heat generated to dissipate easily. All these conditions were maintained to prevent other sources of *error* results that could arise due to interrupted electricity supply, loss of assay integrity due to poor storage and overheating of the Xpert instrument [13].

### Oral HPV testing via traditional PCR and next generation sequencing

Of the 426 samples available for Xpert HPV, there were 407 samples concomitantly tested using traditional SYBR Green Real-Time PCR (Fig. 1). Nineteen samples were deemed to have insufficient volume for traditional PCR.

A pool of primers targeting the L1/L2 (PGMY11/09) region was used. Testing was done at a private laboratory (GenePath Diagnostics, Pune). The primer pool used included those from literature [21] and proprietary primers designed to improve coverage. Positive samples from the first PCR were further processed using a second nested PCR, with typing primers targeting the GP5+/6 + region within the L1 region using modified primers that contained proprietary in-house next generation sequencing (NGS) adapters and indexing tags. The adjusted and pooled amplicons were sequenced using paired-end sequencing by synthesis chemistry (Illumina MiSeq). Genotyping was determined using an in-house developed bioinformatics pipeline. Details of the PCR and NGS methods are provided in the supplementary file.

### Study definitions

We classified Xpert tests results as successful and unsuccessful, as previous studies evaluating the Xpert MTB/RIF have done [13–15]. Samples that generated either negative or positive Xpert results were deemed successful. Samples that generated either *invalid* or *error* results were deemed unsuccessful.

### Statistical analyses

We described successful and unsuccessful results across participant characteristics. Kruskal-Wallis and Fisher’s exact tests were used to compare across continuous and categorical variables, respectively. Statistical significance was set to a p-value of 0.05.

For successful tests, we compared the concordance between the Xpert HPV and SYBR Green Real-Time PCR results using the kappa statistic.

Analyses were performed using Stata 17.0.

## Results

### Successful and unsuccessful xpert tests

Of 426 samples tested on Xpert HPV, 29.8% (n = 127) were unsuccessful. Of these, 78.7% (n = 100) were invalid and the remainder (21.2%, n = 27) error results, respectively (Table 2). All samples with adequate volume (n = 407) were successfully tested on traditional PCR. (Fig. 1).

**Table 2:** Successful and unsuccessful Xpert HPV tests for HPV detection using oral samples in a sample of people living with HIV (HIV) in Pune, India

	Total n (%)	Unsuccessful		Successful	p-value
		Samples reported as invalid n (%)	Samples reported as error n (%)	Samples reported either as positive or negative for HPV n (%)	
**Total N**	426	100 (23.5)	27 (6.3)	299 (70.2)	-
**Procedure of collection and processing** ^**a**^ Procedure 1 Procedure 2	335 (78.6) 91 (21.4)	78 (78.0) 22 (22.0)	21 (77.8) 6 (22.2)	236 (78.9) 63 (21.1)	0.9
**Sex** Female Male	208 (48.8) 218 (51.2)	48 (48.0) 52 (52.0)	14 (51.8) 13 (48.2)	146 (48.8) 153 (51.2)	0.9
**Age** Median age in years (IQR)	40 (34–46)	40 (33–45)	42 (37–45)	40 (34–46)	0.3
**Betel quid or smokeless tobacco use** ^**b**^ Never Ever	257 (60.3) 169 (39.7)	55 (55.0 45 (45.0)	16 (59.3) 11 (40.7)	186 (62.2) 113 (37.8)	0.4
**Smoking history** Never Ever	353 (82.9) 73 (17.1)	86 (86.0) 14 (14.0)	25 (92.6) 2 (7.4)	242 (80.9) 57 (19.06)	0.2
**Alcohol use** Never Ever	279 (65.5) 147 (34.5)	57 (57.0) 43 (43.0)	20 (74.1) 7 (25.9)	202 (67.6) 97 (32.4)	0.1
**Lifetime multiple sexual partners** ^**c**^ No Yes	312 (73.2) 114 (26.8)	73 (73.0) 27 (27.0)	22 (81.5) 5 (18.5)	217 (72.6) 82 (27.4)	0.6
**Oral sex** ^**d**^ No Yes	374 (87.8) 52 (12.2)	92 (92.0) 8 (8.0)	25 (92.5) 2 (7.4)	257 (85.9) 42 (14.1)	0.2
**Self identify as MSM** ^**e**^ No Yes	415 (97.4) 11 (2.6)	100 (100.0) 0 (0.0)	26 (96.3) 1 (3.7)	290 (97.0) 9 (3.0)	0.1
**Suspected OPMD** ^**f**^ No Yes	345 (81.0) 81 (19.0)	78 (78.0) 22 (22.0)	23 (85.2) 4 (14.8)	244(81.6) 55 (18.4)	0.7
**Time updated CD4 counts (cells/mm** ^**3**^ **)** < 500 >= 500 Missing	199 (46.7) 221(51.9) 6 (1.4)	47 (47.0) 50 (50.0) 3 (3.0)	13 (48.2) 14 (51.8) 0 (0.0)	139 (46.5) 157 (52.5) 3 (1.0)	0.7

IQR: Interquartile range (25th quartile to 75th quartile)

^a^**Procedure 1**: oral sample collected using oral rinse only. Testing suspension prepared after double centrifugation of oral rinse; **Procedure 2**: oral sample collected using cytobrush scraping of buccal mucosa + oral rinse. Testing suspension prepared by pooling suspension prepared from oral rinse after double centrifugation + cytobrush fluid

^b^**Betel quid**: Locally available form is called paan (areca nut + slaked lime + condiments, wrapped in a betel leaf used with or without the addition of chewing tobacco); **Smokeless tobacco use**: use of tobacco forms that are not burned. Locally available forms are khaini (tobacco + slaked lime); gutka (tobacco + areca nut + slaked lime); mishri (roasted powdered tobacco); snuff; paan masala

^c^**Multiple sexual partners**: ≥1 lifetime sexual partner

^d^**Oral sex**: given oral sex in their lifetime

^e^**MSM**: Men who have sex with men

^f^**OPMD**: Oral potentially malignant disorders

The median age of participants that contributed oral samples tested on Xpert HPV was 40 years (IQR: 34 to 46), with 51.2% (n = 218) of the samples collected from biological males. More than a quarter of the samples (26.8%, n = 114) were from PLHIV that reported multiple sexual partners in their lifetime. Only 12.2% (n = 52) of the samples came from PLHIV that reported having given oral sex in their lifetime. None of the participants that contributed samples had received any HPV vaccinations.

Successful and unsuccessful tests were not statistically significantly different across Procedure 1 and Procedure 2, or any other variables. (Table 2)

### Agreement of successful Xpert HPV and traditional PCR results

Of 299 successful Xpert HPV tests, there were 286 samples correspondingly tested using traditional PCR. (Fig. 1)

Only one sample was identified as HPV positive by Xpert, resulting in an HPV positivity of 0.3% (i.e., 1/299). Traditional PCR identified 27 samples as HPV positive, resulting in a positivity of 9.4% (i.e., 27/286). (Fig. 1)

The kappa statistic was 0.11, indicating poor agreement between Xpert HPV and traditional PCR results. (Table 3).

**Table 3:** Concordance estimates between Xpert HPV and traditional PCR

Xpert® HPV	SYBR Green Real-Time PCR	
**Agreement %**	94.8%	
**Kappa statistic** **(95% Confidence Interval)**	0.11 (-0.09, 0.31)	
	**SYBR Green Real-Time PCR positive (n)**	**SYBR Green Real-Time PCR negative (n)**
**Xpert HPV Positive (n)**	1	0
**Xpert HPV Negative (n)**	15	270

Concordance was calculated only on those samples that were usable by the Xpert® HPV and had been concomitantly tested using conventional PCR

## Discussion

Approximately a third of the oral samples tested on Xpert HPV had unsuccessful results. Additionally, for successful tests, there was poor agreement between Xpert HPV and traditional PCR results. Our findings indicate that Xpert HPV may not be useful as a screening tool for HPV detection using oral samples among PLHIV.

Estimates for the proportions of unsuccessful Xpert HPV tests using cervical fluid and anal fluid are not available. However, our proportion of unsuccessful tests (29.8%) is higher than those reported for Xpert MTB/RIF across different studies (5–11%) [13–15, 22, 23]. Furthermore, neither SAC nor PCC failures were significantly reduced with a change in oral sample collection methods i.e., the proportion of invalid and error results remained comparable before and after changing oral sample collection methods. These findings suggest that potential oral particulate matter did not contribute significantly to unsuccessful tests, as had been hypothesized.

Most of the unsuccessful tests were due to invalid results. We hypothesize that oral samples even when cytobrush scrapings were included may not consistently have the adequate number of human cells required by Xpert HPV SAC reagents. Moreover, despite ensuring uninterrupted electricity supply, proper assay storage, and adequate sample volume for all tests, the proportion of error results in our study was higher than that recommended by the Xpert manufacturer (i.e., 6.3% vs. 5%) [14]. Error results could also be due to poor sample preparation (e.g., sample could be too viscous) causing tube pressure to increase beyond the acceptable pressure limit. Although the same two laboratory technicians were used throughout the study, we cannot completely rule out improper sample preparation as a cause for error results.

The Xpert HPV limit of detection for different HPV genotypes varies between 10 (for HPV16, 18, 31, 33, 45, 51, 59) to 30 (for HPV 66) HPV DNA plasmid copies per PCR reaction [24]. The low positivity observed for Xpert HPV could be secondary to HPV viral load levels being below these limits of detection in oral samples. Moreover, the comparative traditional approach employed a nested PCR, which may have increased its sensitivity for HPV detection [25], further reducing the agreement with Xpert HPV results.

Our study has several limitations that merit mention. The Xpert HPV is standardized for cervical fluid samples and not oral samples [8]. Though it has been successfully tested using anal fluid samples [10, 11], functionally, the oral cavity constitutes a more dynamic environment relative to the cervix or anal canal (i.e., due to regular ingestion of food, water, salivary secretions, etc.). This dynamicity may itself present a challenge with using oral samples, as exfoliated cell concentration and HPV viral loads could be variably affected by voluntary (i.e., eating, drinking, etc.) and involuntary (salivary secretion) processes. While the time and instructions for oral sample collections were consistent for participants, we were unable to control for variations in voluntary and involuntary processes. We did not retest or recollect samples when an error or invalid result was reported, as recommended by Cepheid [8]. Retesting or recollecting oral samples may have improved the performance of Xpert HPV. Future studies should evaluate the extent to which Xpert HPV performance can be improved with retests and recollections of oral samples. However, in care settings with a high number of people seeking care, retests and recollections may not be practicable. Further, we did not quantify HPV viral loads in oral samples, precluding us from drawing definite conclusions about the association between low viral loads and low Xpert positivity. We did not document the codes for the error results when samples were processed [8, 20]. Therefore, we are unable to identify the exact source (i.e., compromised tube integrity, high tube pressure, overheating, etc.) for them. The files (.gxx files) that contained this information were found to be corrupted when we tried to access them later and could not be retrieved even with the assistance of Cepheid representatives. Lastly, as mentioned, HPV is associated with oropharyngeal cancers. Using a tonsillar brush [26] instead of an oral cytobrush may have improved Xpert positivity.

Based on our findings, we believe that currently Xpert HPV has limited utility for oral HPV screening using oral samples among PLHIV. Otimization of oral sample collection methods may reduce SAC and PCC failures, but the issue of poor concordance with traditional PCR methods remains. However, most samples were successfully tested, and one sample was correctly identified to be HPV positive. This leads us to believe that the performance of Xpert HPV using oral samples could be improved. More research to improve the performance of Xpert HPV and evaluation of its utility for oral samples among PLHIV is required.

## Electronic supplementary material

Below is the link to the electronic supplementary material.

Supplementary Material 1

## Data Availability

Many of the participants in the study have disclosed their HIV status only to the study staff and their treating physician, apart from their spouses or close family members. Thus, the datasets are not publicly available. However, researchers can contact the corresponding author if they are interested in using these data.

## Abbreviations

PLHIV: People living with HIV
HPV: Human papillomavirus
hrHPV: High risk HPV
OPSCCs: Oropharyngeal squamous cell carcinomas
PCR: Polymerase chain reaction
ISH: In-situ hybridization
SAC: Sample adequacy control
PCC: Probe check control
LMICs: Low- and middle-income countries
ART: Antiretroviral therapy (ART)
BJGMC-SGH: Byramjee Jeejeebhoy Government Medical College – Sassoon General Hospitals
CRS: Clinical Research Site
NIH: US National Institutes of Health
VQA: Virology Quality Assurance
OPMD: Oral potentially malignant disorder
NGS: Next generation sequencing
MSM: Men who have sex with men
SLT: Smokeless tobacco
