# Supplementary material for: Challenges with the use of Xpert HPV as a screening tool for oral HPV among people living with HIV (PLHIV): experiences from Pune, India
Source: BMC Infect Dis. 2023 Apr 17;23:233. doi: 10.1186/s12879-023-08210-2 (PMC10108491; doi:10.1186/s12879-023-08210-2)
Supplement: Supplementary file 1 — Supplementary Material 1 [file 12879_2023_8210_MOESM1_ESM.pdf]

Supplementary file

Details of traditional PCR and Next-generation sequencing methods

a. **Reagents detail** - Platinum Taq DNA polymerase (Invitrogen, Cat No. 10966034), HotStarTaq DNA polymerase (Qiagen, Cat No. 203205), dNTP (deoxyribonucleotide triphosphate) 25 micromolar (mM) (Lucigen, Cat No. D59104).

   [Note - Invitrogen PCR buffer and $MgCl_2$ are provided by Invitrogen along with Platinum Taq DNA polymerase].

b. **Thermal cycling conditions, primers used and model of PCR machine** - A nested PCR approach targeting the L1 gene was used. The outer PCR was carried out using an equimolar mixture of each PGMY09 and PGMY11 primer pool [1] at a concentration of 80 nanomolar (nM) each, which generated a 450 base pairs (bp) product. This product was used as a template for the inner PCR. The inner PCR was carried out using custom tagged GP5+ and GP6+ primers [1] at a final concentration 200 nM each, which generated a 150 bp product. The tagged inner PCR products were used for library generation where specific barcodes were incorporated for each sample. The library was sequenced on an Illumina MiSeq or a NextSeq next generation sequencer in a shared run using the Illumina V2 2*250 (MiSeq) or Mid-output 2*150 (NextSeq) paired-end sequencing by synthesis chemistry. The resulting fastq raw read files were aligned against HPV DNA database using in-house bioinformatics pipeline and read counts generated were analysed for presence/absence and type of HPV DNA in each sample.

   The outer PCR was carried out at 20 microlitre (µl) reaction volume, with 1X Invitrogen PCR buffer, 4 mM MgCl2, 0.2 mM dNTP and a blend of two polymerases, 0.5U of Platinum Taq DNA polymerase (Invitrogen) and 0.5U of HotStarTaq DNA polymerase

(Qiagen). The outer PCR was carried out on Veriti 96-well thermal cycler (Applied Biosystems) with initial incubation at 37°C, initial denaturation at 95°C for 15 min, followed by 35 cycles of denaturation at 95°C for 20 sec, annealing at 56.5°C for 30 sec, extension at 72°C for 1 min. The amplified outer PCR products were diluted 100 times with ultrapure nuclease free PCR grade water and used as template for inner PCR.

For the inner PCR, custom tagged GP5+ and GP6+ primers [1] were used at a final concentration of 200 nM each. The inner PCR was carried out at 20ul reaction volume with 1X invitrogen PCR buffer, 4 mM $MgCl_2$, 0.2mM dNTP, 0.5mg/ml BSA, and a blend of two polymerases, 0.5U of Platinum Taq DNA polymerase (Invitrogen) and 0.5U of HotStarTaq DNA polymerase (Qiagen). The inner PCR was carried out on Veriti 96-well thermal cycler (Applied Biosystems) with initial incubation at 37°C, initial denaturation at 95°C for 15 min, followed by 8 cycles of denaturation at 95°C for 20 sec, ramp up annealing from 40°C to 72°C for 30 sec, extension at 72°C for 30 sec, followed by 27 cycles of denaturation at 95°C for 20 sec and extension at 72°C for 1 min.

c. **Housekeeping gene**: No housekeeping gene was used. An approach using unique mapped vs total reads was used to calculate the % HPV reads from sequencing data.

Reference

1. Gravitt, P. E., Peyton, C. L., Alessi, T. Q., Wheeler, C. M., Coutlée, F., Hildesheim, A., Schiffman, M. H., Scott, D. R., & Apple, R. J. (2000). Improved amplification of genital human papillomaviruses. Journal of clinical microbiology, 38(1), 357–361. https://doi.org/10.1128/JCM.38.1.357-361.2000
